# Adherence to Post-endovascular Aneurysm Repair Surveillance According to the Local Galway University Hospital Protocol and Its Impact on Outcomes and Complications (2020–2024)

**DOI:** 10.7759/cureus.112253

**Published:** 2026-07-08

**Authors:** Ahsan Jamil, Abdul Basit, Shaheer Ahmed, Stewart Walsh, Sherif Sultan, Muhammad Tubassam, Mahmoud Alway, Doireann Joyce

**Affiliations:** 1 Department of Vascular Surgery, Galway University Hospital, Galway, IRL; 2 Department of General Surgery, Jinnah Hospital, Lahore, Lahore, PAK; 3 Department of Surgery, Galway University Hospital, Galway, IRL

**Keywords:** angiography, endovascular aneurysm repair (evar), non-adherent, patients, surveillance

## Abstract

Background

This study aimed to determine adherence to post-endovascular aneurysm repair (EVAR) surveillance according to the local Galway University Hospital, Ireland, protocol and assess its impact on outcomes and complications.

Methodology

This retrospective study was conducted at Galway University Hospital, Ireland, including 294 patients who underwent EVAR between January 2020 and December 2024. Data were retrieved from theater logs, radiology Picture Archiving and Communication System/Radiology Information System, clinic letters, and vascular registry. Patients were categorized as adherent, partially adherent, or non-adherent based on attendance within scheduled surveillance windows at three months, six months, 12 months, and annual follow-up.

Results

The mean age was 76 ± 8 years, and 82.0% of patients were male. Full adherence to surveillance was observed in 73.1% patients, while 18.0% were partially adherent and 8.8% were non-adherent. Any endoleak increased significantly with declining adherence (19.5% vs. 24.5% vs. 34.6%, p = 0.012), as did Type I endoleak (p = 0.003). Post-EVAR rupture increased from 0.5% in adherent patients to 3.8% in non-adherent patients (p = 0.032). Aneurysm-related mortality was significantly lower in adherent patients compared with partial and non-adherent patients (1.9% vs. 7.5% vs. 7.7%, p < 0.001). Re-intervention rates did not differ significantly.

Conclusions

Adherence to post-EVAR surveillance according to the local Galway University Hospital, Ireland, protocol was associated with lower complication rates and better aneurysm-related outcomes, while reduced adherence was associated with higher endoleak, rupture, and mortality rates.

## Introduction

Endovascular aneurysm repair (EVAR) has been increasingly used over the past 20 years to treat infrarenal abdominal aortic aneurysms (AAAs) in favor of open aneurysm repair [1]. While the immediate complications of EVAR are low, there is concern about the long-term durability of EVAR with high rates of secondary intervention and a cumulative incidence of 14% at five years [2]. Furthermore, endoleak is the most common complication following EVAR, occurring in up to 25% of patients and may progress to late rupture of the aorta with an incidence of 1.5% [1,3]. Thus, EVAR requires active surveillance to detect and correct late endograft complications [2]. Currently, the Society for Vascular Surgery practice guidelines advocate lifelong surveillance using CT imaging or color duplex ultrasound at one and 12 months in the first year after surgery, followed by annual imaging, with the aim of timely reintervention to prevent late aneurysm rupture [3].

Regular surveillance after EVAR involves a protocol-based approach using imaging techniques such as CT angiography, duplex ultrasound, and plain radiography, depending on institutional practice and risk stratification [4]. These surveillance strategies are designed to ensure graft integrity and aneurysm sac stability, as well as to detect complications requiring reintervention. As such, compliance with surveillance protocols is essential to maximize the safety and longevity of EVAR. However, there are challenges with post-EVAR surveillance adherence. Several studies have documented a decrease in adherence over time, with a substantial number of patients not attending scheduled surveillance appointments and/or not completing recommended imaging studies [5]. Suboptimal compliance could lead to late detection of endoleaks, failure to detect device complications, increased risk of secondary interventions in emergencies, and possibly worse clinical outcomes [6]. The association between surveillance adherence and clinical outcomes has been increasingly recognized. Adherence may be associated with earlier detection of complications, reduced catastrophic late complications, and better opportunity for planned reintervention if needed [7]. By contrast, non-adherence may lead to more complications, greater aneurysm-related events, and poor outcomes [8]. However, results have not been consistent, and local data are valuable for assessing institutional practices. As surveillance practices vary between institutions, adherence to local protocols may be important [9]. Local Galway University Hospital (GUH) protocol-driven surveillance may include surveillance schedules for imaging and clinical follow-up to detect complications following EVAR. Assessment of adherence to this protocol and its association with outcomes may offer clues to the effectiveness of surveillance programs and opportunities for quality improvement [10].

EVAR is the most common method of repair for many patients with abdominal aortic and iliac aneurysms [11]. EVAR has a lower perioperative morbidity than open surgery; however, the long-term success of EVAR requires post-procedural surveillance to detect endoleaks, expansion of the aneurysm sac, migration of the stent, and late rupture. Our local GUH surveillance protocol involves cross-sectional imaging at three months, six months, 12 months, and yearly thereafter [12]. There is a degree of variability in the adherence to post-EVAR surveillance in clinical practice. This can lead to delayed diagnosis of significant complications and adversely affect long-term results [13]. This study was undertaken to evaluate adherence to the local GUH post-EVAR surveillance protocol and to explore the association between surveillance attendance and clinical outcomes.

The objective of this study was to evaluate adherence to the local GUH post-EVAR surveillance protocol among patients who underwent endovascular aneurysm repair between 2020 and 2024. The study aimed to evaluate adherence to the local GUH post-EVAR surveillance protocol and assess its association with clinical outcomes, including endoleak, aneurysm sac enlargement, re-intervention, post-EVAR rupture, and aneurysm-related mortality.

## Materials and methods

This retrospective study conducted at GUH included all EVAR cases performed between January 1, 2020, and December 31, 2024. A total of 294 patients who underwent EVAR during the study period were included. All EVAR cases were included in a consecutive fashion. EVAR patients for AAA or iliac artery aneurysm (IAA) were included. Thoracic endovascular aortic repair, non-aneurysmal disease, and incomplete core clinical and surveillance data were excluded from analysis.

Data sources

Variables were sourced from theater logs, radiology pictures and reports (Picture Archiving and Communication System/Radiology Information System), clinic letters, and vascular registry data. Demographics, aneurysm and comorbidity data, EVAR procedural data, and follow-up surveillance data were recorded. Surveillance adherence was assessed according to the local GUH post-EVAR protocol, which included follow-up imaging at three months, six months, 12 months, and annually thereafter. Patients were classified according to attendance at scheduled post-EVAR surveillance visits under the local GUH protocol. Adherent patients attended all required follow-ups at three months, six months, 12 months, and annually thereafter. Partially adherent patients missed one or more scheduled visits but had at least one documented follow-up or imaging assessment. Non-adherent patients had no documented post-EVAR surveillance follow-up or imaging after the index procedure. Endpoints included compliance with surveillance protocol, development of endoleak, sac enlargement, graft-related events, reintervention, aneurysm-related events, and death. Endoleak was defined as persistent blood flow outside the endograft but within the aneurysm sac on follow-up imaging. Endoleaks were classified as Type I, II, III, IV, or V according to the source of flow. Sac enlargement was defined as an increase in aneurysm sac diameter of ≥5 mm compared with the previous post-EVAR measurement. Graft-related events included graft migration, kinking, limb occlusion, stenosis, component separation, or graft infection. Aneurysm-related mortality was defined as death due to aneurysm rupture, EVAR-related complications, graft failure, or emergency aneurysm-related re-intervention. Three categories of surveillance adherence to the local GUH protocol were created, namely, adherent, partial adherent, and non-adherent, based on attendance during the CT angiography surveillance time periods (at three months, six months, 12 months, and every year after). Surveillance adherence to the local GUH protocol following EVAR was the primary outcome. Secondary outcomes were the effects of surveillance adherence on complications, reintervention, aneurysm sac behavior, and clinical outcomes.

Data analysis

Data were entered and analyzed using SPSS version 26.0 (IBM Corp., Armonk, NY, USA). Categorical variables were reported as counts and percentages (n/N (%)). Continuous variables were presented as mean ± standard deviation or median (interquartile range) as appropriate. Differences between the three adherence groups were assessed using a 3 × 2 chi-square test (one p-value per outcome). The relationship between adherence and complications or outcomes was tested, with p-values ≤0.05 being significant.

## Results

Data were collected from 294 patients, with a mean age of 76 ± 8 years. The cohort was predominantly male (241, 82.0%), while females comprised 53 (18.0%) patients. Median aneurysm size was 5.8 cm. Most patients underwent EVAR for AAA (266, 90.5%), while iliac aneurysms accounted for 28 (9.5%) cases. Hypertension was the most common comorbidity (214, 72.8%), followed by ischemic heart disease (121, 41.2%) and chronic kidney disease (48, 16.3%) (Table 1).

**Table 1:** Baseline demographic and procedural characteristics (n = 294).

Variable	Value
Age (years), mean ± SD	76 ± 8
Male sex, n (%)	241 (82.0)
Female sex, n (%)	53 (18.0)
Median aneurysm size (cm)	5.8 ± 0.8
Abdominal aortic aneurysm	266 (90.5)
Iliac aneurysm	28 (9.5)
Hypertension	214 (72.8)
Ischemic heart disease	121 (41.2)
Chronic kidney disease	48 (16.3)

Multiple stent graft platforms were used, with Medtronic being the most frequently utilized device (86, 29.3%), followed by Gore (74, 25.2%) and Cook (61, 20.7%). Regarding surveillance adherence, the majority of patients were fully adherent to the local GUH protocol (215, 73.1%), while 53 (18.0%) were partially adherent and 26 (8.8%) were non-adherent (Table 2).

**Table 2:** Stent graft types and surveillance adherence status.

Variable	n (%)
Stent graft type	
Medtronic	86 (29.3)
Gore	74 (25.2)
Cook	61 (20.7)
Jotec	39 (13.3)
Endologix	34 (11.6)
Surveillance adherence	
Adherent	215 (73.1)
Partial adherent	53 (18.0)
Non-adherent	26 (8.8)

Any endoleak increased from 42 (19.5%) in adherent patients to 13 (24.5%) in partially adherent and nine (34.6%) in non-adherent patients (p = 0.012). Type I endoleak also increased significantly across groups (4.7% vs. 7.5% vs. 7.7%, p = 0.003). Post-EVAR rupture was uncommon but showed significantly higher frequency with reduced adherence (0.5% vs. 1.9% vs. 3.8%, p = 0.032). Re-intervention rates were low and did not differ significantly between groups (p > 0.05) (Table 3).

**Table 3:** EVAR-related complications by surveillance adherence. EVAR-related complications were compared across surveillance adherence groups using the chi-square test. A p-value ≤0.05 was considered statistically significant. EVAR = endovascular aneurysm repair

Outcome	Adherent, n (%)	Partially adherent, n (%)	Non-adherent, n (%)	P-value
Any endoleak	42 (19.5)	13 24.5)	9 (34.6)	0.012
Type I endoleak	10 (4.7)	4 (7.5)	2 (7.7)	0.003
Re-intervention	12 (5.6)	2 (3.8)	2 (7.7)	>0.05
Post-EVAR rupture	1 (0.5)	1 (1.9)	1 (3.8)	0.032

Aneurysm-related mortality increased from four (1.9%) in adherent patients to four (7.5%) in partially adherent and two (7.7%) in non-adherent patients (p < 0.001). Sac enlargement also demonstrated a significant increase across adherence groups (6.5% vs. 13.2% vs. 19.2%, p = 0.021). Secondary intervention rates did not differ significantly (p = 0.28) (Table 4).

**Table 4:** Clinical outcomes and mortality by surveillance adherence. Clinical outcomes and mortality were compared among adherent, partially adherent, and non-adherent patients using the chi-square test. A p-value ≤0.05 was considered statistically significant.

Outcome	Adherent, n (%)	Partially adherent, n (%)	Non-adherent, n (%)	P-value
Aneurysm-related mortality	4 (1.9)	4 (7.5)	2 (7.7)	<0.001
Sac enlargement	14 (6.5)	7 (13.2)	5 (19.2)	0.021
Secondary intervention required	12 (5.6)	2 (3.8)	2 (7.7)	0.28

Rates of any endoleak, Type I endoleak, post-EVAR rupture, and aneurysm-related mortality all showed progressive increases from adherent to partially adherent and non-adherent groups, supporting a clear association between reduced surveillance compliance and worse aneurysm-related outcomes (Table 5).

**Table 5:** Trend analysis of adverse outcomes with reduced surveillance adherence. Data are presented as n (%). Comparisons between surveillance adherence groups were performed using the chi-square test. EVAR = endovascular aneurysm repair

Outcome	Adherent, n (%)	Partially adherent, n (%)	Non-adherent, n (%)
Any endoleak	42 (19.5%)	13 (24.5%)	9 (34.6%)
Type I endoleak	10 (4.7%)	4 (7.5%)	2 (7.7%)
Post-EVAR rupture	1 (0.5%)	1 (1.9%)	1 (3.8%)
Aneurysm-related mortality	4 (1.9%)	4 (7.5%)	2 (7.7%)

## Discussion

This study showed that there is a clinically relevant correlation between compliance with the local EVAR surveillance protocol and patient outcomes. Patients who underwent planned surveillance imaging experienced fewer endoleaks, EVAR ruptures, and mortality related to the aneurysm compared with patients with partial or no follow-up imaging. A gradient effect between the levels of adherence and the extent of the adverse outcome is suggestive of a dose-response relationship where fully adherent patients have the best outcomes and non-adherent patients the worst outcomes in terms of complications and mortality. Partial adherence was an intermediate risk [13]. Higher endoleak and rupture rates in the non-adherent group may be due to the fact that non-adherent patients may have complications that are detected later than those in the adherent group, which are the focus of structured surveillance. Although establishing causation is not possible, if clinically relevant endpoints are consistent, the necessity for structured post-EVAR follow-up is supported within the long-term treatment of an aneurysm. This study revealed that non-adherence to surveillance is a strong predictor of endoleak [14]. Patients with lower adherence showed a progressive increase in the frequency of any endoleak and Type I endoleak, which indicated that surveillance could be useful in the earlier detection of complications and may prevent the development of clinically relevant events. A similar study published in the past showed that failure to follow surveillance was associated with a delay in intervention and missed device-related complications [15,16].

This is a key point of interest as the percentage of those with poor adherence to surveillance increased among patients who had ruptures after the EVAR procedure. These events of rupture occurred rarely, but the progressive increase in adherence groups implies that poor follow-up may lead to delayed detection of clinically important ruptures [17]. Previous studies have also highlighted that even late ruptures after EVAR are frequently associated with non-detected or non-corrected abnormalities on surveillance. There was also a trend of higher mortality with poorer adherence to treatment for the aneurysm, with statistically significantly lower mortality in the fully adherent group than the partial and non-adherent groups [18,19].

This study has several limitations that should be considered when interpreting the findings. First, its retrospective observational design limits the ability to establish a causal relationship between surveillance adherence and clinical outcomes. Although lower adherence was associated with higher rates of endoleak, rupture, sac enlargement, and aneurysm-related mortality, causality cannot be definitively inferred. Second, the study was conducted at a single tertiary vascular center, which may limit the generalizability of the findings to other institutions with different patient populations, surveillance protocols, imaging practices, and healthcare systems. Third, potential confounding factors such as patient frailty, socioeconomic status, travel distance, health literacy, functional status, and access to healthcare services were not comprehensively evaluated and may have influenced both surveillance adherence and clinical outcomes. Finally, because surveillance data were obtained from institutional records, follow-up investigations or interventions performed at external healthcare facilities may not have been fully captured, potentially leading to underestimation of surveillance adherence or complication rates. Despite these limitations, the study provides valuable real-world evidence regarding the association between adherence to a structured post-EVAR surveillance protocol and long-term aneurysm-related outcomes.

## Conclusions

Adherence to post-EVAR surveillance according to the local GUH protocol was associated with lower rates of endoleak, post-EVAR rupture, sac enlargement, and aneurysm-related mortality. Reduced surveillance adherence showed a clear trend toward worse aneurysm-related outcomes and higher complication burden. These findings support the importance of structured post-EVAR surveillance and suggest that improving adherence may contribute to better long-term outcomes and reduced complications.
